# 

**Published:** 1992-08

**Authors:** 


South West Orthopaedic Club

### WEST OF ENGLAND MEDICAL JOURNAL

**JUNE 1992** **Volume 107(ii)**

Copyright © West of England Medical Journal Ltd. 1991

**CONTENTS**

35 **Editorial**
John Farndon and Paul Goddard

37 **Tuberculosis — the next plague?**
Ben J L Burton

39 **The Great Pretender**

John Potokar

40 **A Peptide Family being Re-united: the Angiotensins Coming in from the Cold**
E C Osborn, A Davenport, A E Goodship, J Campbell Mackenzie

42 **Crossword**
M N Simmonds

43 **The 100th Day Case Disc**
Huw B Griffith

45 **The Mobile Hospitals of the British Red Cross and the Order of St. John and their role in the Rotterdam Typhoid Epidemic 1945**
Lumsden Walker

47 **Factors which predispose elderly patients to develop peptic ulcer disease**
A F Safe

49 **Give us back our 11 days**
Hamish Thomson

50 **Rheumatology and its inception in the South West**
G D Kersley

51 **Sir William Osler, Oxford and "The Open Arms"**
Lord Walton of Detchant

55 **South West Orthopaedic Club**

57 **South West Vascular Surgeons**

60 **South West Surgical Club**

63 **South West Obstetrical and Gynaecological Society**

64 **Crossword: answers**

